# Early infant diagnosis of HIV in Myanmar: call for innovative interventions to improve uptake and reduce turnaround time

**DOI:** 10.1080/16549716.2017.1319616

**Published:** 2017-06-02

**Authors:** Soe Thiha, Hemant Deepak Shewade, Sairu Philip, Thet Ko Aung, Nang Thu Thu Kyaw, Myo Minn Oo, Khine Wut Yee Kyaw, May Wint War, Htun Nyunt Oo

**Affiliations:** ^a^ HIV Unit, International Union Against Tuberculosis and Lung Disease (The Union), Mandalay, Myanmar; ^b^ Department of Operational Research, International Union Against Tuberculosis and Lung Disease (The Union), New Delhi, India; ^c^ Department of Community Medicine, Government T.D. Medical College, Alappuzha, India; ^d^ Department of Operational Research, International Union Against Tuberculosis and Lung Disease (The Union), Mandalay, Myanmar; ^e^ Public Health Laboratory, Department of Medical Services, Ministry of Health and Sports, Mandalay, Myanmar; ^f^ National AIDS Program, Department of Public Health, Ministry of Health and Sports, Nay Pyi Taw, Myanmar

**Keywords:** Early diagnosis/utilization, uptake of EID, turnaround time, HIV-exposed babies, SORT IT

## Abstract

**Background**: In collaboration with the national AIDS program, early infant diagnosis (EID) is implemented by Integrated HIV Care (IHC) program through its anti-retroviral therapy (ART) centers across 10 cities in five states and regions of Myanmar. Blood samples from the ART centers are sent using public transport to a centralized PCR facility.

**Objectives**: Among HIV-exposed babies <9 months at enrolment into IHC program (2013–15), to describe the EID cascade (enrolment, sample collection for PCR, result receipt by mother, HIV diagnosis and ART initiation) and factors associated with delayed (>8 weeks of age) or no blood sample collection for EID.

**Methods**: Retrospective cohort study involving record review. A predictive poisson regression model with robust variance estimates was fitted for risk factors of delayed or no sample collection.

**Results**: Of 1349 babies, 523 (39%) of the babies’ mothers were on ART before pregnancy. Timely uptake of EID (<8 weeks of age) was 47% (633/1349); sample collection was delayed in 27% (367/1349) and not done in 26% (349/1349) babies. Among samples collected (n = 1000), 667 results were received by the mother; 52 (5%) were HIV-infected; among them 42 (81%) were initiated on ART. Median (IQR) turnaround time from sample collection to result receipt by mother and time to initiate ART from result receipt by mother was 7 (4,12) and 8.5 (6,16) weeks, respectively. Mothers not on ART before pregnancy and distance of ART center from PCR facility (more than 128 km) were the risk factors of delayed or no sample collection.

**Conclusions**: Improving provision of ART to mothers (through universal ‘test and treat’) is urgently required, which has the potential to improve the timely uptake of EID as well. Interventions to reduce turnaround times, like point of care EID testing and/or systematic use of mobile technology to communicate results, are needed.

## Background

Globally, out of 36.7 million people living with HIV, 1.8 million were children [1]. Most of the transmission to children younger than 15 years was from their mothers: majority acquired during perinatal period and the rest through breast feeding [2]. Despite the dramatic progress in prevention of mother to child transmission (PMTCT) program, there were still 150 000 new infections among children in 2015 [1].

Half of the babies acquiring HIV infection from the mother die within two years without treatment, of which 33% die within the first year [3]. Therefore, once a baby is born to a mother living with HIV (HIV-exposed baby), it is imperative that the baby is tested for HIV early and if found positive (HIV-positive baby), initiated on antiretroviral therapy (ART). In low-and middle-income countries, only 42% of HIV-exposed babies receive timely virologic testing and only 23% of HIV-positive receive ART [4]. These significant gaps of HIV diagnosis and treatment coverage may lead to rapid progression of disease and/or death.

The World Health Organization (WHO) recommended early infant diagnosis (EID) involves testing of HIV-exposed babies between six and eight weeks, particularly using a polymerase chain reaction (PCR) technology [5,6]. There is evidence of missed opportunity for EID from African countries. Long turnaround time (TAT) for EID was associated with non-receipt of the result by the mother [7,8]. This may have an implication on delayed ART initiation and further clinical management in HIV-positive babies.

The HIV epidemic in Myanmar is concentrated among key populations and the prevalence in adult population is 0.54%. There were 210 000 people living with HIV (9700 deaths due to HIV) in 2014, of them 11 000 were children [9]. The International Union Against Tuberculosis and Lung Disease (The Union) has been implementing an integrated HIV care (IHC) program in Myanmar in collaboration with the national AIDS program (NAP) since 2005. In March 2012, The Union, in collaboration with Fondation Merieux, provided logistic and technical support to set up a PCR facility (HIV DNA and RNA viral load) at the Public Health Laboratory (PHL) in Mandalay, the third largest city in Myanmar [10]. The IHC program provides ART and EID through its ART centers.

Until now there has been no systematic assessment of EID in Myanmar and EID monitoring is not part of routine monitoring and evaluation of the NAP. Findings from the study would feed into the improvement of the program and also act as a baseline for studies in the future.

Therefore, in this operational research, we aimed to study EID among HIV-exposed babies eligible for a PCR test (age <9 months at enrolment) under the IHC program, Myanmar (2013–15). ***Specific objectives*** were to (1) describe their socio-demographic, clinical, and programmatic characteristics; (2) describe the EID cascade, including timely uptake of EID (<8 weeks of age), TAT from sample collection to result receipt by mother, and time to initiate treatment after result receipt by mother; and (3) determine the factors associated with delayed (>8 weeks of age) or no sample collection.

## Methods

### Study design

This was a retrospective cohort study of EID cascade involving record review of routinely collected data. EID cascade included enrolment into IHC programme, sample collection for PCR, receipt of PCR results by mother, HIV diagnosis, and treatment initiation.

### Setting

#### General setting

Myanmar is situated in South-East Asia and has an estimated population of 51 million, of whom 70% live in rural areas. It has 15 states and regions. They are administratively divided into districts and further into 412 townships/sub townships [11].

#### IHC program

The IHC program works through a public-private-patient-partnership model and provides HIV care based on national guidelines [12]. It is involved in 15 ART centers across ten cities in five states and regions. Among them, twelve ART centers have a pediatrician and provide EID. Data of HIV-exposed babies is maintained as a separate database in all these centers except in Sagaing and Kalaw.

#### EID under IHC

WHO-recommended EID services were available by the end of 2012 [5]. When an HIV-exposed baby is delivered, he/she is enrolled at nearest IHC. Both mother and baby are expected to visit within six weeks of birth for timely EID. After the enrollment visit, baby is continued on zidovudine or nevirapine syrup (prescribed from hospital of birth) if necessary, and started on cotrimoxazole preventive therapy.

A blood sample collection date (dry blood spot /DBS) for DNA PCR or frozen plasma in an ice box for RNA PCR) and a follow-up date, one month later, is allotted. After sampling by a trained technician, entry of the sample collection date is made and the sample is sent to PHL by public transportation (once/twice a week). PCR is routinely run within two weeks of sample receipt at PHL, subject to receipt of sufficient number of samples. The PCR result is sent back to the respective ART center, again by public transportation, usually within one week. Public transportation was used for paper-based communication of results because of limited availability of internet at the ART centers. The Union logistic team ensures that the sample and test result reach the respective destination. The Union also supports technical trainings for blood sample collection and transportation at laboratories in ART centers.

The results are reported to the mother/care giver during the scheduled follow-up visit (generally one month after sample collection). If the result is negative, a routine follow-up visit at three-monthly intervals is advised. Antibody tests are done around the age of nine months and 18 months.

**Figure 1.**
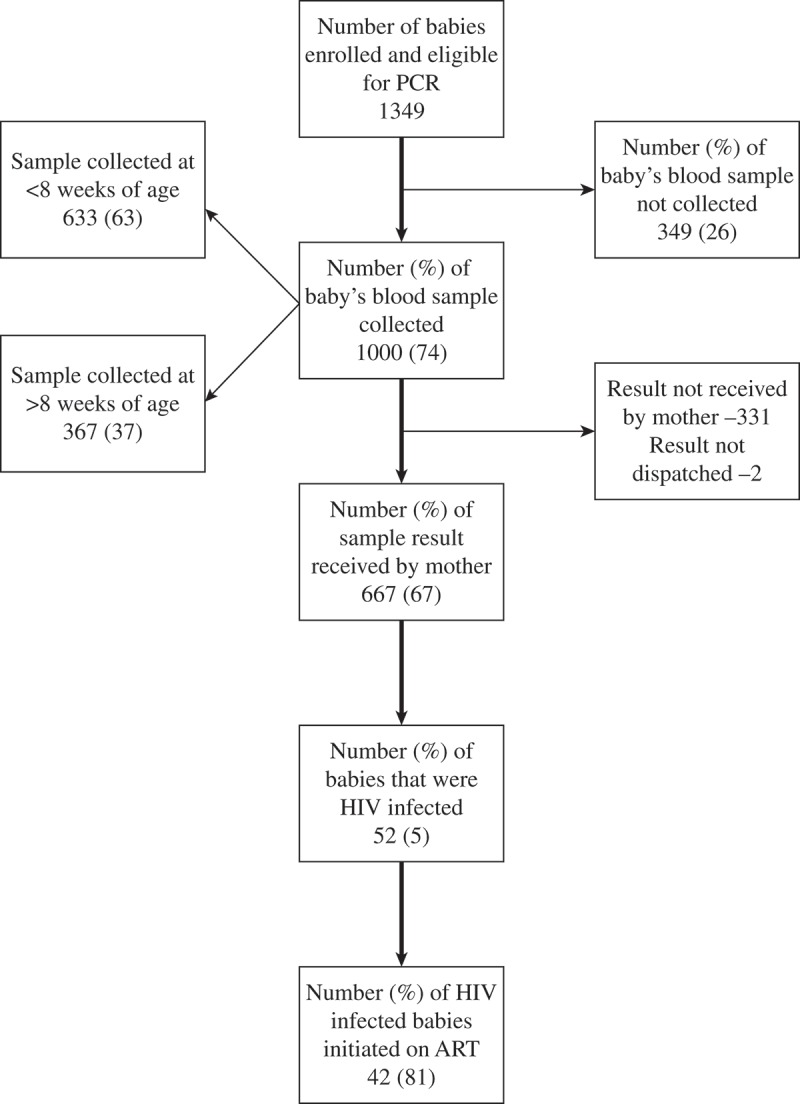
Flow chart for early infant diagnosis cascade among HIV-exposed babies eligible for PCR test (age <9 months at enrolment) under integrated HIV care program, Myanmar, 2013–2015. Note: HIV: human immunodeficiency virus; PCR: Polymerase chain reaction test used for early infant diagnosis of HIV; ART: anti-retroviral therapy.

If a baby gets a positive result (PCR result if <9 months age; antibody test result if 9–18 months age), the doctor orders baseline investigations and refers the baby to the pediatric ART clinic for ART initiation. A repeat PCR test is ordered for confirmation if the initial test is an antibody test. EID cascade is shown in Figure 1.

Enrolled babies are provided a unique identifier and information is recorded in their treatment cards at the ART center and the electronic PCR database at PHL. The ART center data are routinely single entered in the electronic database of the IHC program with a systematic built-in mechanism for data quality checks. The HIV status of the baby in the electronic records is the final diagnosis by the pediatrician.

### Patient population

All HIV-exposed babies aged <9 months at enrollment into IHC program between January 2013 and December 2015 at the ten out of the12 ART centers providing EID, Myanmar, were included in the study. Two ART centers in Sagaing and Kalaw were excluded, as a separate database of HIV-exposed babies was not maintained.

### Data variables, sources of data, and data collection

Between October and November 2016, records of HIV-exposed babies were extracted from the electronic databases at ART centers and PHL and merged using the unique identifier. They were tracked from enrolment to PCR testing (at ≤9 months); and among the above cohort, from diagnosis to treatment initation if HIV positive.

In addition to key socio-demographic and clinical characteristics (Tables 1 and 2), data variables extracted included IHC code (unique identifier); date of birth, enrolment, sample collection, result receipt by mother, and ART initiation; result of PCR test; whether confirmatory test done (for HIV positive); and distance of ART center from the baby’s township and PHL. The mother visit date immediately after the result dispatch was considered as the date of receipt of result by mother. Distance was calculated using google maps (www.google.com./maps).

**Table 1.** Socio-demographic characteristics of HIV-exposed babies eligible for a PCR test (age <9 months at enrolment) under the integrated HIV care program, Myanmar, 2013–2015.

Characteristics		n	%
Total		1349	100
Sex of baby			
	Male	655	49
	Female	637	47
	Not recorded	57	4
Age of mother at enrolment			
in years	<18	3	<1
	18–24	218	16
	25–34	600	44
	≥35	169	13
	Not recorded	359	27
Mother’s marital status			
	Single mother	10	1
	Married	727	54
	Widow	40	3
	Divorced/separated	13	1
	Not recorded	559	41
Mother’s education status			
	Literate	702	52
	Not literate	239	18
	Not recorded	408	30
Mother’s employment status			
	Employed	308	23
	Unemployed/homemaker	461	34
	Not recorded	580	43

HIV: human immunodeficiency virus; PCR: Polymerase chain reaction test used for early infant diagnosis of HIV.

**Table 2.** Clinical and programmatic characteristics of HIV-exposed babies eligible for a PCR test (age <9 months at enrolment) under integrated HIV care program, Myanmar, 2013–2015.

Characteristics		n	%
Total		1349	100
Mother’s baseline CD4			
cells/mm^3^	<100	102	8
	100–349	403	30
	≥350	404	30
	Not recorded	440	32
Mother’s pre-delivery			
treatment status	On ART before pregnancy	525	39
	On PMTCT	281	21
	Not on ART and PMTCT	543	40
Mode of delivery			
	NSVD	449	33
	AVD	3	<1
	LSCS	795	59
	Not recorded	102	8
Place of delivery			
	Hospital	1111	82
	Private	3	<1
	Home	135	10
	Not recorded	100	8
Programmatic factors		N	Median (IQR)
Distance of ART center from patient’s township in kilometers		1349	11 (3,49)
Distance of PCR facility from patient’s ART center in kilometers		1349	128 (3, 279)

Note: HIV: human immunodeficiency virus; PCR: Polymerase chain reaction test used for early infant diagnosis of HIV; ART: anti-retroviral therapy; PMTCT: prevention of mother to child transmission; NSVD: normal spontaneous vaginal delivery; AVD: assisted vaginal delivery; LSCS: lower section caesarian section; IQR: Inter quartile range.

### Analysis and statistics

Data extracted in MS EXCEL was imported into EpiData analysis software (version 2.2.2.183, EpiData Association, Denmark) for descriptive and unadjusted analysis. Multivariable adjusted analysis was done using STATA (version 12.1 STATA Corp, USA).

Frequency and proportion was used to summarize categorical variables. Age and CD4 count (continuous variables) were categorized as described in Tables 1 and 2. Median (inter-quartile range, IQR) was used to summarize distance. 1-Kaplan-Meier curve was used to describe the cumulative proportion of babies tested at different ages. In the absence of sample collection, each baby was right censored at nine months of age.

Timely uptake of EID was defined as sample collection before eight weeks of age. Outcome of interest was the composite of delayed (≥8 weeks of age) or no sample collection. Unadjusted analyses were performed to assess the factors associated with delayed or no sample collection. Variables with a p-value of <0.2 in the unadjusted analysis were fitted in predictive multivariable model: poisson regression with robust variance estimates (enter method). For modelling, distance was categorized based on the median value. Variables with high multicollinearity (mother employment and literacy status), assessed using variance inflation factor, were excluded. HIV-exposed babies with missing variables were excluded from regression analysis: complete case analysis was done. Unadjusted and adjusted relative risks (RR) were reported with 95% confidence intervals (CI).

### Ethics

Ethics approval was obtained from the Ethics Advisory Group, The Union, Paris, France; and Ethics Review Committee at Department of Medical Research, Yangon. Permission to conduct the study was taken from NAP Myanmar. As this study involved analysis of secondary programmatic data, waiver of informed consent was sought and obtained from the ethics committees.

## Results

Of the 1461 HIV-exposed babies, 92 babies were more than nine months of age at enrollment, and the date of birth was missing for 20 babies. Hence, 1349 babies were included in this analysis. The socio-demographic, clinical, and programmatic characteristics have been summarized in Tables 1 and 2. Of 1349 babies, 655 (49%) were males, and 525 (39%) of the babies’ mothers were on ART. The median distance (in km) of the ART center from the patient’s township and the ART center from PHL was 11 (3,49) and 128 (3,279) respectively (Table 2).

**Figure 2.**
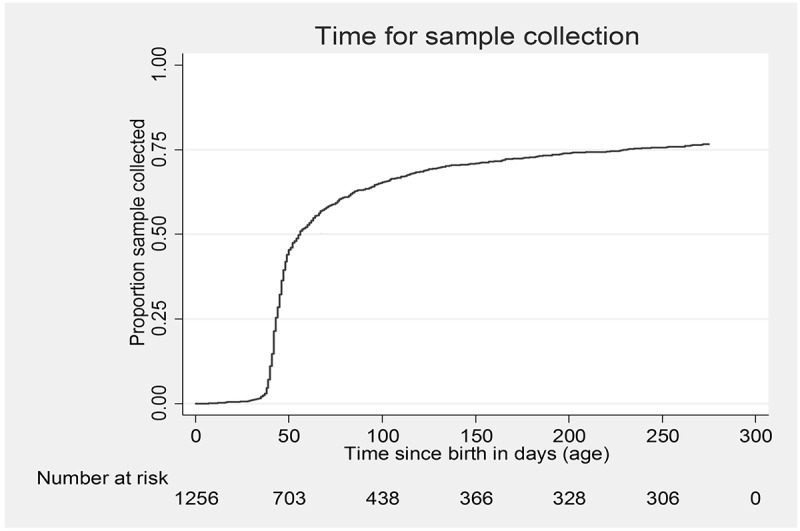
Cumulative proportion of sample collected at different ages among HIV-exposed babies eligible for PCR test (age <9 months at enrolment) under integrated HIV care program, Myanmar, 2013–2015 (N = 1256)*. Note: Number at risk in the figure indicates those who are eligible for PCR and not tested at the given time*1-Kaplan Meier curve; 93 HIV-exposed babies who belonged to the patient population (age <9 months at enrolment) but were excluded from this analysis due to non-availability of dates.

Of 1349 babies, timely uptake of EID was 47% (633/1349); sample collection was delayed in 27% (367/1349) and not done in 26% (349/1349) babies. The timely uptake of EID was 47% every year from 2013 to 2015. Among samples collected (n = 1000), 667 results were received by the mother and 52 (5%) were HIV-infected. Forty two (81%) were initiated on ART among HIV-infected. (Figures 1 and 2).

Median (IQR) age at enrolment and at sample collection was 1.4 (0,7) and 6.7 (7,11) weeks, respectively. Median (IQR) TAT from sample collection to result receipt by mother, and median time to initiate treatment after result receipt by mother was 7 (4,12) and 8.5 (6,16) weeks, respectively (Table 3).

**Table 3.** Median age at enrolment /blood collection and turnaround time in EID cascade among HIV-exposed babies eligible for PCR test (age <9 months at enrolment) under integrated HIV care program, Myanmar, 2013–2015.

Age /time in weeks	n*	Median	IQR
Median age at enrollment	1349	1.4	0.0, 7.0
Median age at blood collection	1000	6.7	6.0, 10.7
Turnaround time to mother receiving result from blood collection	677	7.0	4.0, 12.0
Turnaround time to start ART from result receipt by mother (among HIV-infected)	42	8.5	5.8, 15.9

*Includes patients who were available and respective dates were recorded.

Note: EID: Early infant diagnosis; HIV: human immunodeficiency virus; PCR: Polymerase chain reaction test used for early infant diagnosis of HIV; ART: anti-retroviral therapy; IQR: Inter quartile range.

On unadjusted analysis, mother being non-literate, unemployed, on PMTCT only or neither on PMTCT nor on ART; mothers undergoing normal vaginal delivery, delivery at home; distance of ART center from baby’s township; and distance of ART center from PHL were the risk factors associated with delayed or no sample collection (Table 4).

**Table 4.** Factors associated with delayed (≥8 weeks of age) or no blood sample collection among HIV-exposed babies eligible for PCR test (age <9 months at enrolment) under integrated HIV care program, Myanmar, 2013–2015.

Variable	Type	Evaluated ** (n)	Delay/not collected n (%)	RR (95%CI)	aRR (95%CI)**
Total		1349	716 (53)	–	–
**Socio-demographic**					
Sex of baby	Female	637	331(52)	1.0(0.9,1.1)	1.0(0.8,1.1)
	Male	655	328(50)	Ref.	Ref.
Age of mother in years	<18	3	0(0)	–	–
	18–24	218	107(49)	1.1(0.8,1.3)	1.0(0.8,1.3)
	25–34	600	266(44)	1.0(0.8,1.2)	0.9(0.8,1.2)
	≥35	169	75(44)	Ref.	Ref.
Mother’s marital status	Single mother	10	4(40)	1.0(0.4,2.3)	–
	Married	727	275(38)	Ref.	–
	Widow	40	11(28)	0.7(0.4,1.2)	–
	Divorced/separated	13	7(54)	1.4(0.9,2.4)	–
Mother’s education status	Literate	702	255(36)	Ref.	–
	Not literate	239	175(73)	2.0(1.8,2.3)*	–
Mother employed	Yes	308	99(32)	Ref.	–
	No/Homemaker	461	190(41)	1.3(1.0,1.6)*	–
**Clinical factors**					
Mother’s baseline	<100	102	46(45)	1.2(0.9,1.5)	–
CD4 cells/mm^3^	100–349	403	155(39)	1.0(0.8,1.2)	–
	≥350	404	161(40)	Ref	–
Mother’s pre-	On ART	281	121(43)	Ref.	Ref.
delivery treatment	On PMTCT	525	161(31)	1.4(1.2,1.7)*	1.4(1.1,1.8)*
status	None	543	434 (80)	2.6(2.3,3.0)*	1.8(1.5,2.2)*
Mode of delivery	NSVD	449	275(61)	1.4(1.3,1.6)*	1.1(1.0,1.3)
	AVD	3	2(67)	–	–
	LSCS	35	23(66)	Ref	Ref
Place of delivery	Hospital	1111	533(48)	Ref.	Ref.
	Private	3	1(33)	–	–
	Home	135	94(70)	1.5(1.3,1.7)*	1.2(0.9,1.4)
**Programmatic factors**					
Distance from ART	≥11 km	719	424(59)	1.3(1.2,1.4)*	1.0(0.9,1.2)
center to patient residence	<11 km	630	292(46)	Ref.	Ref.
Distance from ART	≥128 km	700	517(74)	2.4(2.1,2.7)*	2.2(1.8,2.7)*
center to laboratory	<128 km	649	199(31)	Ref.	Ref.

*statistically significant, p < 0.05; ** Adjusted RR, complete case analysis (415 cases excluded), using poisson regression (enter method); factors with unadjusted p < 0.2 were included in model; mother employment/literacy status, were excluded due to high collinearity.

Note: HIV: human immunodeficiency virus; EID: early infant diagnosis; ART: anti-retroviral therapy; PMTCT: prevention of mother to child transmission; NSVD: normal spontaneous vaginal delivery; AVD: assisted vaginal delivery; LSCS: lower section caesarian section; km: kilometer; RR: Relative risk.

On adjusted analysis (415 HIV-exposed babies were excluded from adjusted analysis due to missing variables), mother being on PMTCT only [RR 1.4, 95% CI 1.1-1.8] or neither on PMTCT nor on ART [RR 1.8, 95% CI 1.5-2.2] had higher risk of delayed or no sample collection when compared to mothers on ART before pregnancy. Distance of ART center from PCR facility, more than 128 km, was a risk factor for delayed or no sample collection when compared to a distance <128 km [RR 2.2, 95% CI 1.8-2.7] (Table 4).

## Discussion

We found that more than half of HIV-exposed babies enrolled into IHC programme in Myanmar did not get timely EID. Turnaround times were long. Timely uptake of EID decreased if babies’ mothers were not on ART before pregnancy and their ART center was far away from the PCR facility.

### Strengths and limitations

This is the first study from Myanmar to systematically study EID on a large scale. All the HIV-exposed babies enrolled into IHC were included. The study was operational in nature, utilizing existing resources without additional funding. Hence, the findings are representative and present a true picture of the program. We used google maps for calculating the distance [13], as this was not routinely recorded in program.

There were some limitations in the study. We could not look at the cascade in depth. Dates for sample receipt and testing at PHL were not routinely recorded. Date of result dispatch at PHL was available but could not be used because if a baby was tested more than once, then the date of dispatch was recorded for the most recent result. We assumed that the immediate date of follow-up visit in records after result dispatch was the date of result receipt by the mother. However, there are chances that the mother followed up on the designated date but the results were not available. We could not assess the independent effect of mother’s literacy and employment status on delayed or no sample collection due to high collinearity. Due to the secondary nature of the data, information on variables was missing in some records. This was also partly because many mothers were not enrolled into IHC.

### Key findings

There were many policy relevant findings. First, the IHC program needs to address the low uptake of EID among HIV-exposed babies within eights weeks of age. Our findings of timely testing are comparable with the global WHO figures (43%) and studies conducted elsewhere in Africa and Asia. The timely uptake ranged from 62% in Namibia, 40% in Thailand and 34% in Haiti [4,14,15]. Around 22% of the HIV-exposed babies were enrolled after eight weeks of birth (data not shown). This coupled with the prolonged TAT could explain the poor timely uptake of EID. Non-availability of a trained technician at the ART center on all days may also be factor for poor timely uptake. More than 80% of HIV-exposed babies were delivered at public hospital facilities, poor timely uptake of EID despite this could be explained by a poor referral linkage system with EID facilities at those hospitals. Significant attrition (22–70%) was reported post testing in Senegal, Uganda, Cambodia, and Namibia; similar attrition was also seen in our study (Figure 1) [16].

Second, the median TAT (seven weeks) from sample collection to result receipt by mother was higher than three to five weeks reported from studies conducted in African and Caribbean countries, three to seven weeks in Nigeria, five weeks in Ethiopia, and five to six weeks Uganda. However, it was lesser than seven to ten weeks in Mozambique, 39 weeks in rural Tanzania and 13 weeks in rural Zambia [8,14,17–21]. Long TAT may be due to dependence on public transportation for transfer of samples to PHL and communication of test results to the ART center, sample waiting time at PHL and the distance between ART center and PHL (median 128 km).

Third, timely uptake increased if mothers were on ART before pregnancy similar to the finding in Ethiopia [20]. Even if the mother was enrolled and receiving PMTCT before delivery, she had a 40% higher risk for missing timely sample collection for her baby when compared to mother receiving ART before pregnancy. A mother who was enrolled in IHC and also receiving ART before pregnancy must have been on regular follow-up and thus aware of the need for early testing for her baby when compared to other mothers living with HIV. This is programmatically relevant as only two-fifths of mothers were on ART before pregnancy (Table 1).

Fourth, while the distance between ART and PCR facility was associated with delayed or no sample collection, distance between ART center and baby’s township was not. Therefore, we can assume that delayed or no EID uptake among HIV-exposed babies who enrolled in time was due to the long distance between the ART center and PHL.

Fifth, more than 80% of HIV positive babies in the cohort were initiated on ART. This indicated good linkage of EID services and Pediatric clinic (providing ART for HIV-positive babies). The program, however, needs to address the long time taken to initiate ART. We did not have sufficient numbers of patients initiated on ART (n = 42) in our cohort to study the risk factors for the long time taken to initiate ART.

### Recommendations for policy and practice

This study has some important recommendations, not just for the IHC program but for the country’s HIV/AIDS prevention and control program as well. Firstly, WHO recommended universal ‘test and treat’ for people living with HIV should be urgently implemented at the field level in Myanmar [22]. This will ensure reduced transmission to baby and increase the chances of timely EID if mother is on ART as shown in our study. Second, point of care technology providing PCR testing should be introduced in ART centers with high caseloads because this technology for EID can shorten the TAT and has standard quality against conventional methods [23]. Third, a short messaging service has been started in 2016 by PHL and the national health laboratory in Yangon. Similar innovative ideas to communicate EID results, like global package radio service printers [17], and use of courier services for transporting samples may be implemented systematically. Fourth, EID services should be integrated at multiple entry points – antenatal care clinics, immunization clinics, and other baby care centers and not just be available in ART centers [14,23]. Fifth, resources for hiring and training additional technicians for pediatric blood sampling should be considered. Finally, routine data collection within the IHC program needs to improve considering the large number of records with missing data (Tables 1 and 2). The program should systematically record the date of receipt of sample and date of testing at PHL. Date of result dispatch should be recorded for each sample.

Similar operational research, including dates at various steps at PHL, may be repeated in future to test the effectiveness of interventions to increase timely uptake of EID and reduce TATs. To explore the perspectives of mothers and program personnel, qualitative research is recommended for barriers in utilizing and implementing EID respectively. In this cohort, around one third of mothers did not receive the results after sample collection. Hence, assessment of factors affecting delayed TAT from sample collection to result receipt by mother and/or non-receipt of results is recommended.

## Conclusion

This operational research identified low timely uptake of EID, high turnaround times in EID cascade and factors associated with delayed or no EID in IHC program, Myanmar. The program needs to look at innovative approaches to address these if they are to achieve the target of 90-90-90 by 2020 (90% of all people living with HIV will know their HIV status, 90% of all people with diagnosed HIV infection will receive sustained ART. 90% of all people receiving ART will have viral suppression) [23].

## Paper context

This is the first study seeking the timeliness of early infant diagnosis(EID) in HIV-exposed babies analyzed in large cohort at Myanmar. EID using polymerase chain reaction(PCR) can diagnose HIV infection and initiate treatment early leading to reduce morbidity and mortality. Low uptake of EID and delay in treatment initiation after diagnosis were observed in this study. This may require immediate policy attention in the field level of HIV care and treatment centers implementing EID services in Myanmar.
